# Processing of emotional words measured simultaneously with steady-state visually evoked potentials and near-infrared diffusing-wave spectroscopy

**DOI:** 10.1186/1471-2202-11-85

**Published:** 2010-07-27

**Authors:** Leonie Koban, Markus Ninck, Jun Li, Thomas Gisler, Johanna Kissler

**Affiliations:** 1 Department of Psychology, University of Konstanz, Universitätsstrasse 10, 78457 Konstanz, Germany; 2 Department of Physics, University of Konstanz, Universitätsstrasse 10, 78457 Konstanz, Germany; 3 Swiss Center for Affective Sciences, University of Geneva, Rue des Battoirs 7, 1205 Geneva, Switzerland

## Abstract

**Background:**

Emotional stimuli are preferentially processed compared to neutral ones. Measuring the magnetic resonance blood-oxygen level dependent (BOLD) response or EEG event-related potentials, this has also been demonstrated for emotional versus neutral words. However, it is currently unclear whether emotion effects in word processing can also be detected with other measures such as EEG steady-state visual evoked potentials (SSVEPs) or optical brain imaging techniques. In the present study, we simultaneously performed SSVEP measurements and near-infrared diffusing-wave spectroscopy (DWS), a new optical technique for the non-invasive measurement of brain function, to measure brain responses to neutral, pleasant, and unpleasant nouns flickering at a frequency of 7.5 Hz.

**Results:**

The power of the SSVEP signal was significantly modulated by the words' emotional content at occipital electrodes, showing reduced SSVEP power during stimulation with pleasant compared to neutral nouns. By contrast, the DWS signal measured over the visual cortex showed significant differences between stimulation with flickering words and baseline periods, but no modulation in response to the words' emotional significance.

**Conclusions:**

This study is the first investigation of brain responses to emotional words using simultaneous measurements of SSVEPs and DWS. Emotional modulation of word processing was detected with EEG SSVEPs, but not by DWS. SSVEP power for emotional, specifically pleasant, compared to neutral words was reduced, which contrasts with previous results obtained when presenting emotional pictures. This appears to reflect processing differences between symbolic and pictorial emotional stimuli. While pictures prompt sustained perceptual processing, decoding the significance of emotional words requires more internal associative processing. Reasons for an absence of emotion effects in the DWS signal are discussed.

## Background

Emotionally significant stimuli automatically attract attention and have been shown to be processed preferentially in comparison to neutral stimuli. This effect is apparent for both unpleasant stimuli, such as angry faces, snakes or humiliation scenes, and in pleasant material like erotica or baby faces, as investigated in behavioral experiments, in studies using EEG, and in fMRI [1-3]. Functional MRI experiments indicate increased BOLD responses during presentation of intrinsically unpleasant stimuli as well as aversively conditioned stimuli in different brain areas, including - perhaps surprisingly - even primary visual areas [4-6]. Using near-infrared spectroscopy (NIRS), two recent studies [7,8] reported increased metabolic activity in the occipital lobes for emotional, and in particular pleasant, compared to neutral pictures.

EEG event-related potential (ERPs) recordings found an enhanced early posterior negativity (EPN) between 200 ms and 300 ms after stimulus onset and an increased late positive potentials (LPPs) around 500 ms for emotional compared to neutral pictures from the International Affective Picture Set (IAPS, [9]) [10-12]. Further, eliciting steady-state visual evoked potentials with oscillatory flickering IAPS pictures also leads to enlarged signal amplitudes for emotional pictures, pointing at higher activity in primary visual areas (e.g., [13,14]).

Enhanced sensory processing of emotional stimuli is presumed to be mediated by feedback projections from the basal nucleus of the amygdala to the striate cortex and extrastriate, secondary visual and associative processing areas [15]. The amygdala is an almond-shaped structure in the anterior medial temporal lobe strongly involved in emotion processing [16]. Through this so-called "reentrant processing" the amygdala may therefore enhance neural responses to emotionally relevant stimuli [15].

Even purely symbolic stimuli such as words seem to be subject to the effects of enhanced visual processing and attention allocation, as shown in studies investigating event-related EEG potentials (ERPs) to emotionally significant words (see [17] for a review). Even in very early time windows emotionally significant words affect visual processing, as indicated by enlarged P2 s at parietal electrodes (around 180-250 ms after stimulus onset) for pleasant [18] or both pleasant and unpleasant words [19]. Similarly, an enhanced early posterior negativity (EPN) has been found at around 250 ms for emotional in comparison to neutral words [20-23].

Regarding late (> 300 ms) ERP effects, several studies found enhanced LPPs for both pleasant and unpleasant compared to neutral words (e.g., [23-25]). However, in some studies only pleasant words induced an enhanced LPP [19,20,26], although early effects were found for both pleasant and unpleasant words. Due to specific elaborative associative processing, later effects may have been restricted to pleasant stimuli [19,20]. Evidence for deeper associative processing particularly of words with pleasant emotional content also comes from two studies probing the startle eye blink response [19,27].

Event-related functional magnetic resonance imaging also revealed increased BOLD responses to emotionally relevant words in limbic areas and visual cortex [28,29]. A recent study of single word reading found the largest effects during reading of pleasant words, as reflected in activation increase in both occipital visual areas and the amygdala. The increase in activation of these areas were correlated, in line with the model of re-entrant processing [30].

While it is becoming clear that emotionally significant words benefit from transient amplification of visual processing, it is an open question whether this enhanced activation is also seen in prolonged and sustained electrophysiological responses of the visual cortex, such as steady-state visual evoked potentials (SSVEPs), for which emotion effects have previously been found using IAPS picture stimuli [13,14]. The present study addresses this issue combining electrophysiological and non-invasive optical measures of brain activity in response to the presentation of flickering word stimuli.

Diffusing-wave spectroscopy (DWS; also called diffuse correlation spectroscopy, DCS) is an emerging optical technique for the non-invasive detection of functional brain activity. The contrast mechanism of DWS is based on the dynamics of scatterers within tissue - such as erythrocytes - that is enhanced e.g. by the increase of regional blood flow associated with the vasodilatation induced by electrical activity [31-35]. The first entirely non-invasive applications of DWS to brain function detection [32,34] focused on the human motor cortex stimulated by finger tapping tasks, which elicit large functional signals. However, such tasks interfere with systemic perfusion changes accompanying functional cortical activation.

Recent technical advances, which have significantly improved the signal-to-noise ratio of the DWS experiment [36], have allowed for detection of functional DWS signals from much smaller, activated areas located more deeply in the cortex, such as the human primary visual cortex where functional activation can be elicited without changes of systemic perfusion. For steady-state visual stimulation with 30 s full-field flickering, functional DWS signal changes of 3.0-3.8% were found [33] which can be traced back to the functional increase of rCBF of 60-80% measured with PET [37].

In addition to these slow DWS signals, Li et al. [35] reported that short (8.2s) visual stimulation blocks elicit a transient slowing of cortical dynamics localized on the left hemisphere of the visual cortex which is not explained by the current models of neurovascular coupling [38]. While NIRS and DWS signals yield complementary information on neurovascular coupling, the larger functional signal obtainable with DWS makes this technique attractive for studies in neuroscience.

So far, DWS has not been applied to experiments examining very subtle stimulus differences such as the emotional connotation of words, but it could provide additional and complementary information on hemodynamic changes in response to emotional stimuli.

Combining DWS and EEG requires an experimental paradigm that provides sustained stimulation over several seconds and at the same time a number of trials sufficient for a satisfying signal-to-noise ratio of the EEG response. Steady-state visual evoked potentials (SSVEPs) offer both these features. They are oscillatory, nearly sinusoidal scalp potentials in response to a repeated visual stimulation presented at frequencies of 4-6 Hz or higher [39,40]. Their main advantage is a very good signal-to-noise ratio for moderate stimulation times, and a sharp peak at the stimulation frequency in the power spectrum of the signal [39]. Further, SSVEPs provide a unique possibility to study a well-defined and controllable electrophysiological brain response to prolonged stimulation. Hence, SSVEPs are ideally suited for investigating simultaneous EEG and metabolism-based techniques like optical measurements of brain activity. Larger SSVEP amplitudes were found during viewing of emotional in comparison to neutral pictures [13,14], but so far no study has investigated SSVEP modulation during processing of emotional words. As detailed above, the neural dynamics underlying processing of emotional words are in many ways similar to the processing of emotional pictures and lead to increased attention and resource allocation. However, the emotional significance of words lies in their symbolic nature, which is not primarily conveyed by external stimulus characteristics, but by the activated associations. Therefore, during prolonged and sustained stimulation, emotion modulation for words may differ from those of pictures.

Here, we investigate SSVEPs and DWS signals evoked by pleasant, unpleasant, and neutral words. In line with previous literature [17,20,24,28], we reasoned that emotional (pleasant and unpleasant) words would attract more attention than neutral words. Most SSVEP studies show higher SSVEPs for attended than for unattended stimuli [41,42]. In parallel, extant SSVEP studies on affective picture processing [13,14] have reported larger responses to emotional than to neutral pictures. However, two recent studies suggest that, under certain circumstances, SSVEP power can be reduced rather than increased by attention, e.g. when narrowing the spatial focus of endogenous attention [43] and depending on the oscillating brain network [44]. So far, no study examined SSVEP modulation by emotional words, precluding specific directional predictions. Based on previous research showing differential processing of emotional and neutral stimuli, we hypothesized that SSVEP power as well as the DWS signal would be different for emotional compared to neutral words.

## Methods

### Participants

Twelve students from the University of Konstanz (six women and six men, mean age 22.7 years) with normal or corrected-to-normal vision and no history of neurological or psychiatric illness volunteered to participate in the experiment. All participants were right-handed and native German speakers and gave informed consent. The study protocol was approved by the University's Ethical Review Board. The optical data of two participants had to be discarded because of low photon count rate, thus only ten optical data sets were used for the DWS data analysis.

### Stimuli

30 different German nouns previously rated by an independent sample on the dimensions of valence, arousal and concreteness [21] were used as stimulus material. According to their valence ratings, the stimuli were grouped into three emotion categories: nouns with pleasant, neutral and unpleasant valence. One-way repeated measures ANOVAs confirmed that the categories differed significantly in valence (*F*(2,27) = 981.85, *p *< 0.0001), with Tukey tests showing differences between all categories (neutral vs. positive, *p *= 0.0001, neutral vs. unpleasant, *p *= 0.0001, pleasant vs. unpleasant, *p *= 0.0001). Differences were also found regarding arousal (*F*(2,27) = 79.70, *p *< 0.0001), with Tukey tests revealing arousal ratings higher for pleasant than for neutral (*p *= 0.0001), and higher for unpleasant than for neutral words (*p *= 0.0001), whereas pleasant and unpleasant words did not differ in arousal (*p *= 0.179). There were no differences between the categories regarding possible confounds such as word length (*F*(2,27) = 0.01, *p *= 0.987), concreteness (*F*(2,27) = 0.03, *p *= 0.970), and word frequency (*F*(2,27) = 0.01, *p *= 0.987). All means and standard deviations for stimulus characteristics are listed in Table 1. Valence and arousal ratings in Table 1 are based on pre-existing normative ratings. Table 2 shows the respective valence and arousal ratings as obtained from the present experimental participants. The actual German stimuli, together with their English translations are given in Table 3.

**Table 1:** Characteristics of stimulus words

	*Valence*		*Arousal*		*Concreteness*		*Word Length*		*Word Frequency*	
	*M*	*SD*	*M*	*SD*	*M*	*SD*	*M*	*SD*	*M*	*SD*
**Unpleasant**	1.69	0.41	5.93	0.82	4.65	0.88	6.40	1.71	194.8	349.7
**Neutral**	4.99	0.10	1.93	0.34	4.75	2.23	6.30	1.34	195.7	152.4
**Pleasant**	8.18	0.38	5.31	0.97	4.83	1.30	6.40	1.71	211.3	232.6

The emotion categories differed only in valence and arousal ratings, but were comparable regarding concreteness, word length and word frequency [21]. M: mean; SD: standard deviation.

**Table 2:** Results of stimulus ratings in the present sample

	*Valence*		*Arousal*	
	*M*	*SD*	*M*	*SD*
**Unpleasant**	2.13	0.53	6.32	1.07
**Neutral**	4.93	0.17	2.09	0.30
**Pleasant**	8.18	0.31	6.26	0.99

The emotion categories differed significantly in valence and arousal ratings. M: mean; SD: standard deviation.

**Table 3:** Stimulus words in the three emotion categories

*Neutral*	*Pleasant*	*Unpleasant*
Apparat (*apparatus*)	Ferien (*holidays*)	Lügner (*liar*)
Stirn (*forehead*)	Freude (*delight*)	Alptraum (*nightmare*)
Begriff (*concept*)	Orgasmus (*orgasm*)	Opfer (*victim*)
Bewohner (*inhabitant*)	Geschenk (*present*)	Elend (*misery*)
Ding (*thing*)	Glück (*happiness*)	Folter (*torture*)
Thema (*theme*)	Liebe (*love*)	Selbstmord (*suicide*)
Geschirr (*tableware*)	Spass (*fun*)	Kerker (*dungeon*)
Inhalt (*content*)	Umarmung (*hug*)	Diktator (*dictator*)
Papier (*paper*)	Kuss (*kiss*)	Krieg (*war*)
Quadrat (*square*)	Vergnügen (*amusement*)	Panik (*panic*)

The stimulus words are listed in German language (as presented in the experiment) and with English translation in parentheses.

### Procedure

**Figure 1:**
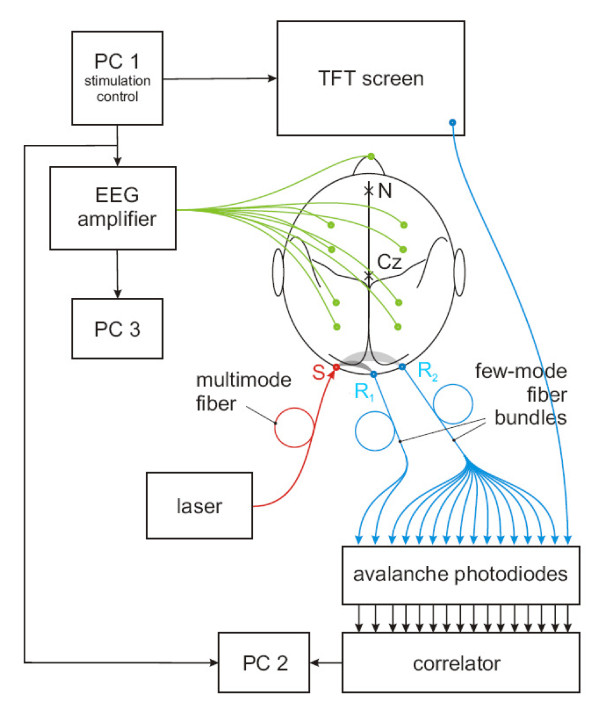
**Experimental setup for simultaneous EEG and DWS measurements**. The stimulation sequence delivered by the thin-film transistor (TFT) screen is driven by a personal computer (PC1) that also delivers the trigger signal for the EEG amplifier. The EEG amplifier records the signals from the 17 scalp electrodes and the reference electrode (green). These data are stored on PC3. PC1 also provides the trigger signal for PC2 which controls the 32-channel USB autocorrelator and stores the DWS data. Light from a diode laser operating at 802 nm is directed via a multimode optical fiber (red) onto the source position S on the scalp over the visual cortex of the subject. Receivers R_1 _and R_2 _(blue), consisting of bundles of few-mode optical fibers, collect photons which have traveled through the gray-shaded, banana-shaped tissue regions. Within both R_1 _and R_2 _the few-mode optical fibers are placed at distances of less than 1 mm in order to detect light intensity fluctuations from equivalent, but statistically independent speckles. Receiver R_1 _(28 fibers) with a large source-receiver distance of 30 mm probes the volume marked in light grey including parts of the visual cortex; receiver R_2 _(3 fibers) with a source-receiver distance of 15 mm probes the superficial layers (scalp and skull, dark grey). Receiver R_3 _records the optical contrast from the TFT screen. N: nasion, Cz: vertex.

After general explanations about the experiment and the electrophysiological and optical recordings, subjects signed an informed-consent form and were asked standardized questions about age, handedness, current or previous psychiatric and neurological illness and in particular epilepsy as potential exclusion factors. After placing the EasyCap and attaching all electrodes, subjects were asked to lie down on the examination couch and the optical fiber bundles were placed at the back of the head. The stimulation was shown on a 17'' TFT computer monitor positioned 40 cm above the subject's head. The experimental setup is shown in Figure 1.

The stimuli were presented in white *Arial *style letters with a size of 72 points on a black background, resulting in a stimulus height of 3.0 cm and width of 7.8-19.7 cm on the screen. In order to detect the exact timing of stimulus on- and offsets with an optical fiber placed at the border of the screen, a white, 2 cm wide, frame was presented together with the words. Each of the 30 different flickering words was presented three times in randomized order, resulting in a total number of 90 trials. Each trial consisted of 8.2 s flickering at a steady-state frequency of 7.5 Hz, i.e., 66.7 ms "On" and 66.7 ms "Off" in each cycle. Trials were separated by a baseline period during which a small red fixation cross was shown in the centre of the otherwise black screen. The duration of the inter-trial baseline period was chosen at random from a flat distribution between 8 and 12 s, in order to avoid synchronization of pulsation with the stimulation. Every 15 trials (after about five minutes), subjects were allowed to have a short break and close their eyes. The whole experimental stimulation took about 30 minutes and was controlled using Presentation software.

After the experiment and after detaching the optical probe and the EEG electrodes, subjects were asked to rate the previously seen words according to their perceived valence and arousal using the Self Assessment Manikin Rating Scale [45] on a different PC for comparison of individual ratings from experimental participants with the a priori ratings.

### EEG recording and processing

Electrophysiological brain activity was recorded with 17 Ag/AgCl scalp electrodes at positions FPz, Fz, FT7, FT8, Cz, C3, C4, TP7, TP8, Pz, P3, P4, P9, P10, Oz, O1, and O2 of the international 10/20 system [46], using an EasyCap for electrode fixation and NEUROSCAN SynAmps amplifier and Acquire386 software. The reference was placed at the tip of the nose and the ground electrode behind the right ear. The EEG signal was high-pass filtered (0.1 Hz, 24dB/oct) online and digitized at a rate of 500 Hz. Impedances were kept well below 5 kΩ. Further, the electrocardiogram as well as horizontal and vertical electrooculograms, were recorded for artifact monitoring.

Offline, EEG data was filtered with a high pass (0.5 Hz, 6 dB) and a 50 Hz notch filter to reduce noise from the unshielded laboratory environment. Eye artifacts were corrected using the topographical correction algorithm implemented in the BESA software [47]. For analysis of the experimental conditions, power spectral densities of the EEG signal over time windows of 8192 ms after stimulus onset were computed by fast Fourier transformation (FFT), using apodization with a cos^2^-window (Hanning window). The resulting frequency spectra at the occipital electrodes O1, Oz, and O2, which were closest to the DWS probe and which cover the typical peak of the SSVEP, were averaged and the steady-state-intensity was extracted by integration over the interval 7.320 - 7.686 Hz.

### DWS measurements

Figure 1 shows the experimental set-up: A diode laser operating at a wavelength of 802 nm was used as a light source. The laser beam was coupled into a multimode optical fiber, which was positioned over the occipital cortex, 15 mm above the Oz electrode. Two receivers collected the emitted light at lateral distances of 30 mm (R_1_) and 15 mm (R_2_) on the left of the source, respectively. While the short-distance optical probe is mainly sensitive to the dynamics within superficial tissue layers (scalp and parts of the skull), receiver R_1 _is additionally sensitive to cerebral blood flow. This setup resulted in a probing of cortical activity approximately 15 mm above and 15 mm on the left relatively to the Oz electrode position. Receivers R_1 _and R_2 _were bundles of 28 and 3 few-mode fibers, respectively; each of the fibers was connected to an avalanche photodiode (APD). From the pulse output of the APDs the temporal autocorrelation function of the fluctuating photon count rate was computed by an autocorrelator. The integration time per measurement and resulting temporal resolution of the DWS measurement was *T' *= 26.2 ms (see Figure 1). Further details on the DWS brain imaging technique and setup can be found in [33,36].

From the bundle-averaged field autocorrelation functions that were calculated separately for receivers R_1 _and R_2_, the decay time *τ*_d _(the actual DWS signal) was calculated. The decay time *τ *_d _serves as an indicator of the scatterer dynamics within the banana-shaped tissue regions spanned by the source and receiver fibers and contains information regarding regional cerebral blood flow changes. Further, the photon count rate *R *(corresponding to the NIRS signal) was measured as the number of detected photons per integration time *T'*.

### Data analysis

#### EEG

The electrophysiological steady-state intensities of the three different experimental conditions (neutral, pleasant and unpleasant words) at the electrode positions O1, Oz, and O2, where the steady-state signal usually peaks and which are closest to the DWS sensor were compared with two-way repeated measures ANOVA.

#### DWS

For each stimulation trial, normalization of the decay times τ_d_(*t*) and photon count rates *R*(*t*) with their respective baseline-averaged values, yielded the relative decay time τ_d,stim_(*t*) and relative photon count rate *R*_stim_(*t*), respectively, as a function of time *t *after stimulation onset. Mean relative decay times ⟨τ_d,stim_⟩ and mean relative photon count rates ⟨*R*_stim_⟩ were obtained by averaging τ_d,stim_(*t*) and *R*_stim_(*t*), respectively, over single stimulation trials.

The mean relative decaytime ⟨τ_d,stim_⟩ (reflecting the cortical blood flow during the stimulation phase relative to the one during the baseline) and the mean relative count rate ⟨*R*_stim_⟩ of all stimulation trials were compared to the baseline condition in order to test the hypothesis that DWS detects functional brain activity. In a second step, *R*_stim_(*t*) and τ_d,stim_(*t*) for each emotional condition were averaged separately and compared with repeated measures ANOVAs.

## Results

### Results of the rating task

ANOVAs on the arousal and valence ratings in the present sample (see Table 2) confirmed the previously obtained results [[21], and see Table 1]: significant differences for the three categories regarding valence (*F*(2, 27) = 682.4, *p *< 0.0001) and arousal (*F*(2, 27) = 79.1, *p *< 0.0001) were found. Tukey tests showed significant differences in rated valence between all categories (*p *< 0.001) and differences in rated arousal between neutral and both categories of emotional words (*p *< 0.001), but not between pleasant and unpleasant words (*p *= 0.988).

### EEG results

**Figure 2:**
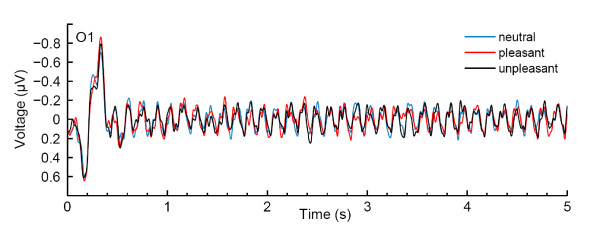
**Grand average evoked potentials for different emotional word categories at electrode O1**. For better readability the SSVEP is cropped after 5 s since there were no major changes in the subsequent time course.

**Figure 3:**
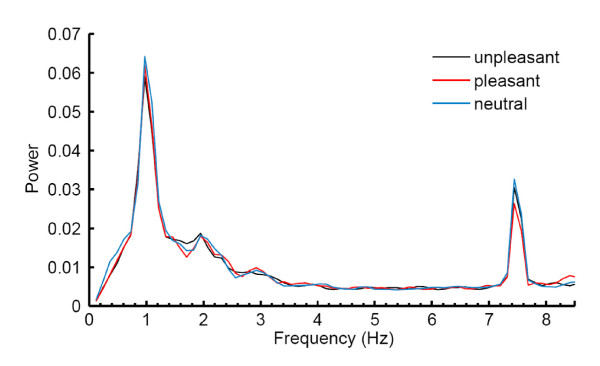
**Grand average of FFT power spectra of the SSVEPs associated with the different emotional word categories at electrode position O1**. Note that this is the average of single-trial power spectra and not the frequency spectrum of the time domain grand average. The evoked peak is seen around 7.5 Hz, corresponding to the frequency of the flickering stimulus. Around 1 Hz, electrophysiological activity is due to heartbeat artifacts.

**Figure 4:**
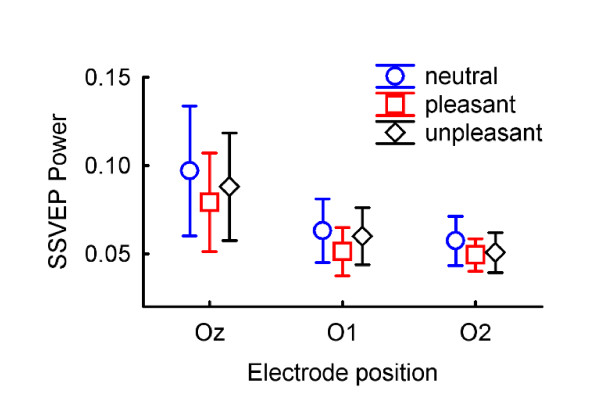
**Average steady-state power for different emotional word categories at electrodes Oz, O1 and O2**. Vertical bars denote standard errors. A significant main effect was found for emotion category, *F*(2, 22) = 3.47, *p *= 0.049.

Steady-state intensities were compared in a two-way repeated measures ANOVA, resulting in a significant main effect for emotional category (*F*(2, 22) = 3.47, *p *= 0.049). Figure 2 depicts the EEG grand average in the time domain (event-related potential) at electrode O1. Figure 3 shows the grand average power spectrum at electrode O1. Time-resolved Gabor filtering [48] was used to investigate whether the pattern of SSVEP emotion modulation changed in the course of the SSVEP stimulation. Since this was not the case, results are not further reported here. There was a trend for electrode position (*F*(2, 22) = 2.75, *p *= 0.086), and also for the interaction between emotional category and electrode position (*F*(4, 44) = 2.34, *p *= 0.070), reflecting a stronger emotion effect at positions Oz and O1 compared to O2 (see Figure 4). To investigate the direction of the main effect for emotional category, planned comparisons with Tukey tests were conducted, revealing differences in SSVEP power only between neutral and pleasant words (*p *= 0.039), with pleasant words eliciting smaller SSVEP power than neutral ones.

### DWS results

**Figure 5:**
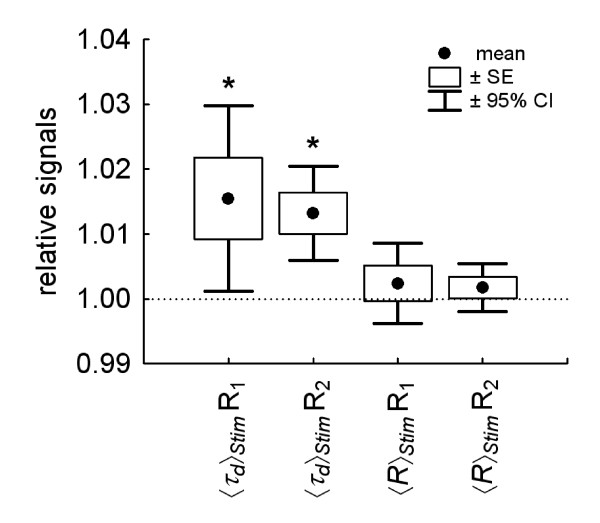
**DWS results: mean baseline-normalized changes of decay time *τ*_*d *_and count rate *R *during stimulation**. Boxes show standard errors and vertical bars denote 95% confidence intervals. R_1_: long-distance, R_2_: short-distance receiver.

**Figure 6:**
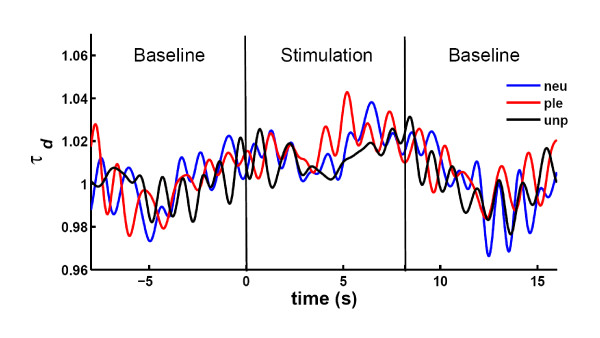
**Grand average time course of the relative decay time *τ*_*d *_measured with the large-distance receiver R_1_**. A low-pass 1 Hz Butterworth filter was applied to facilitate visual inspection of the signal (see also [8]). Emotionally neutral, pleasant and unpleasant values of the presented words are labeled with 'neu' (blue line), 'ple' (red line) and 'unp' (black line), respectively.

Regarding the optical data, *t*-tests revealed significant differences between stimulation and baseline in the mean relative DWS signals, resulting in an increase of 1.55% for the long-distance receiver R_1_, *t*(9) = 2.45, *p *= 0.037, and an increase of 1.32% for the short-distance receiver R_2_, respectively, *t*(9) = 4.14, *p *= 0.003. No stimulation-dependent significant differences were found in the photon count rate for either R_1 _(*t*[9] = 0.86, *p *= 0.411) or R_2 _(*t*[9] = 1.06, *p *= 0.316). All stimulation-dependent changes are shown in Figure 5. Regarding the results of repeated measures ANOVAs on possible effects of emotional word conditions, no differences between word categories were found in the photon count rate or in the decaytime at both source-receiver distances. The low-pass filtered (1 Hz cut-off, 5^th ^order Butterworth) grand averages of the decaytime time course for neutral, pleasant and unpleasant words are presented in Figure 6.

## Discussion

By simultaneously measuring DWS and EEG, the present study aimed to investigate visual cortex activity in response to the presentation of flickering emotional and neutral words. Both methods revealed effects of stimulation, but an emotional modulation was only observed in the SSVEP, not in the DWS signal. DWS showed a statistically significant difference in mean decay time between stimulation and baseline periods for both long- and short-distance receivers R_1 _and R_2_. While the functional signal from the long-distance receiver replicates the previously reported transient slowing of cortical dynamics [35] and extends it to written word stimuli, we find a functional signal also in the data from the short-distance receiver which might arise from residual sensitivity of the short-distance sensor to cortical dynamics, or from increased blood flow in the scalp due to changes in peripheral perfusion induced by the stimulation. No differences in the DWS signals could be detected when comparing the three different emotional word conditions. Given the relatively subtle psychological difference between emotionally positive, negative and neutral words and the modest sample size of ten participants, DWS as implemented in this study may at present not be sensitive enough to detect these small effects. Theoretically, since the present DWS instrumentation allowed recording only from a part of the visual cortex, existing activation differences suggested by some previous fMRI studies [28-30] may show a regional distribution different from the one captured by the present design. The DWS sensors were placed 15 mm above Oz, thus one might argue that they were not measuring activity of the same cortical areas as reflected by the SSVEPs. However, given the limited conductivity of the skull leading to spatial low-pass filtering of the EEG signal, the SSVEPs recorded at the occipital electrodes reflects activation of a larger brain area, probably including the volumes probed by the optical experiment.

Two previous studies using a related optical technique, NIRS, found emotion effects, particularly for pleasant stimuli from the IAPS picture set [7,8]. However, these effects were less clear-cut than corresponding EEG effects. This pattern could result from the lower sensitivity of metabolic contrasts to transient events. Moreover, emotional modulation of visual processing is generally considerably larger for colored pictures than for perceptually much simpler written words [22,49,50]. Thus, future DWS studies of affective processing using pictures from the IAPS set which are perceptually more engaging may well reveal significant effects. Conversely, since no previous NIRS study has investigated signal modulations by emotional words, it is unclear whether a corresponding experiment using conventional NIRS measurement would yield significant results. In the present data, no effect was obtained regarding the photon count rate which is the signal most closely reflecting NIRS. The transmitted light intensity measured by NIRS is proportional to the photon count rate measured in our DWS experiments. The present study found no statistically significant differences in photon count rate due to emotion categories. This could be due to the fact that the DWS photon count rate was measured from the speckle signal, which intrinsically has large fluctuations, and therefore leads to considerably lower signal-to-noise ratio than typical NIRS signals. An additional explanation for the absence of statistically significant functional NIRS signals in our data may be that the photon count rate measured at the laser wavelength of 802 nm is sensitive to changes of total hemoglobin concentration; these are smaller than the functional concentration changes of oxy-hemoglobin that are measured with standard NIRS instruments using two wavelengths located above and below the isosbestic point of hemoglobin. Given these important limitations, the question whether emotional content-dependent differences during word processing can be obtained using blood flow-based measures other than fMRI thus begs further research.

Regarding the SSVEPs, positive words elicited significantly smaller electrophysiological responses than neutral words at occipital electrode positions. The finding of larger differences between positive and neutral than negative and neutral words in emotion word processing is in line with some other previous reports [20,23,26,30,51], but the direction of our finding diverges from results of enhanced SSVEP power for emotional in comparison to neutral IAPS pictures [13,14]. However, to our knowledge, the present study is the first to use a typical SSVEP paradigm to investigate the visual processing of emotional words, which may differ from the processing of emotional pictures.

The higher SSVEP amplitudes for neutral words cannot be due to confounding factors such as word length, frequency, or concreteness, as these factors were matched between conditions. Further, the ratings of valence and arousal of the word stimuli by the participants also confirmed the data previously obtained by Kissler et al. [21], ruling out the possibility that our sample of subjects was different with regard to valence or arousal judgments.

The present results may be accounted for by other factors: First, most previous studies investigating emotion effects on SSVEP amplitudes used a stimulation frequency between 10 and 13 Hz (e.g., [13,52,53]), which might be functionally different from lower frequencies. Investigating different driving frequencies, some authors found enhanced SSVEP amplitudes to attended stimuli for both 12 and 8.6 Hz [41], but the interplay between stimulation frequency and attentional modulation of SSVEPs is not entirely understood yet: In a first systematic investigation of the effects of spatial attention and driving frequency on SSVEPs, Ding and colleagues [44] found higher amplitudes for non-attended stimuli at a stimulation frequency of 9.2 Hz, when a competing attended stimulus was presented to the fovea, but smaller amplitudes when the competing attended stimulus was located in the periphery. Similarly, Chen et al. [43], using a driving frequency of 8.3 Hz and measuring MEG, found a reduction of steady-state visual evoked field amplitude to an attended stimulus when attention was directed to the center of the visual field.

However, it is important to note that these studies did not investigate emotional or exogenous attention but rather endogenous spatial attention. These two forms of attention have partly different underlying mechanisms and effects on visual processing. Whereas the deliberate (endogenous) allocation of attention to certain spatial positions requires top-down control of visual areas by fronto-parietal attention networks, enhanced attention to motivationally salient, emotional stimuli is considered to be automatic and bottom-up, involving the amygdala and its reentrant connections to modulate visual cortex activation [3,54].

Therefore, our results might be due to the specific characteristics of word processing. SSVEPs are responses to stimulation of several seconds and they do not necessarily show the same effects as ERPs, which reflect the immediate first reaction to a stimulus. Regarding emotional picture stimuli, enhanced amplitudes have been observed in ERPs and in SSVEP [10-13,55]. However, words differ from pictures in at least two very important aspects. First, they constitute perceptually relatively simple stimuli, in our case white letters on black ground. On a purely perceptual level, words are far easier to process than colorful photographs of complex scenes, for which visual analysis of all details and relevant aspects should take longer. Letter streams per se are very boring stimuli, as everyone will confirm who has been confronted with a text in an unfamiliar language. The import of a familiar word depends on its meaning, on the associated concepts and associated sensory representations it evokes. This mainly symbolic nature of words is the second and most important aspect distinguishing words from pictures, giving rise to the possibility of different reaction patterns to emotional words than to other, non-symbolic, emotional stimuli. While the initial response to reading an emotional word and seeing an emotional picture as reflected by ERP modulations seems very similar, subsequent sustained activity may diverge. Both emotional pictures and words activate subcortical emotion circuits [56-58]. However, as the emotional response is not caused by the letters on the screen themselves, but their semantic content, it is possible that subsequent stronger processing of emotional relevance takes places at a higher, associative level, which may result in a shift of attention to internal processes rather than external stimulation. This activity in associative cortices is probably not oscillating at the stimulation frequency, but may lead to a decrease of the SSVEP signal due to decreased primary visual processing. Indeed, electrocortical activity enhancements in response to emotional words have been most consistently found at processing stages that reflect activity outside the primary visual cortex [20,21]. Moreover, in line with the possibility of reductions in purely perceptual processing, Herbert et al. [19,27] showed that specifically pleasant adjectives are processed more deeply than neutral ones and that processing of pleasant adjectives leads to a stronger startle reaction in comparison to neutral words, reflecting a more internal focus of attention and higher costs of reorienting attention to external startle cues. From subjective experience, readers know the absorbing effects of exciting novels, leading almost to forgetting of external time and space, while creating an internal world of its own. Therefore, deeper internal processing of pleasant words may be associated with decreased activity in primary visual cortex as reflected by the observed reduced SSVEP amplitudes.

In summary, the present study demonstrates stimulation-driven modulation of both the DWS signal and the SSVEP. The SSVEP, but not the DWS signal, revealed differences due to the emotional content of the words used for stimulation. By showing a relatively smaller SSVEP response to stimulation with pleasant than with neutral words the direction of the emotion effect diverges from previous findings of studies using IAPS pictures. We suggest that this effect reflects specific processing characteristics induced by emotional words.

## Conclusions

This is the first study to concurrently investigate the modulation of diffusing-wave spectroscopy, an optical measure of brain function, and steady state visually evoked potentials (SSVEPs), an electrophysiological measure of brain functioning, by stimulation with trains of flickering positive, negative, and neutral words. Both measures responded to the stimulation as reflected in increased activity as compared to baseline. However, only SSVEPs captured differences elicited by the stimulation's emotional content. Emotional modulation was reflected in a reduction in SSVEP power in response to pleasant as compared to neutral words. While a more pronounced modulation of cerebral activity during processing of pleasant words is in line with previous literature, the reduction of SSVEP power is a new finding, probably due to the symbolic nature of emotional language stimuli, which, similar to emotional pictures, elicits enhanced processing at temporally early stages, but then leads to increased internal processing at the cost of primary sensory activity during prolonged stimulation.

## Authors' contributions

LK designed and programmed the experiment, recorded and analyzed the electrophysiological data and wrote the manuscript. JK and TG contributed to the design of the study and to the writing of the manuscript. TG; JL and MN designed and set up the DWS experiments. MN set up the synchronization of EEG and DWS and was in charge of the DWS recordings. JL analyzed the optical data. JK and LK set up the EEG in the DWS environment. All authors read and approved the final manuscript.

## References

[B1] Brosch T Sander D Pourtois G Scherer KR Beyond fear: rapid spatial orienting toward positive emotional stimuli Psychological Science 2008 19 362 370 10.1111/j.1467-9280.2008.02094.x 18399889

[B2] Dolan RJ Emotion, cognition, and behavior Science 2002 298 1191 1194 10.1126/science.1076358 12424363

[B3] Vuilleumier P How brains beware: neural mechanisms of emotional attention Trends in Cognitive Sciences 2005 9 585 594 10.1016/j.tics.2005.10.011 16289871

[B4] Bradley MM Sabatinelli D Lang PJ Fitzsimmons JR King W Desai P Activation of the visual cortex in motivated attention Behavioral Neuroscience 2003 117 369 380 10.1037/0735-7044.117.2.369 12708533

[B5] Knight DC Smith CN Stein EA Helmstetter FJ Functional MRI of human Pavlovian fear conditioning: patterns of activation as a function of learning Neuroreport 1999 10 3665 3670 10.1097/00001756-199911260-00037 10619663

[B6] Lang PJ Bradley MM Fitzsimmons JR Cuthbert BN Scott JD Moulder B Nangia V Emotional arousal and activation of the visual cortex: an fMRI analysis Psychophysiology 1998 35 199 210 10.1017/S0048577298001991 9529946

[B7] Herrmann MJ Huter T Plichta MM Ehlis A-C Alpers GW Mühlberger A Fallgatter AJ Enhancement of activity of the primary visual cortex during processing of emotional stimuli as measured with event-related functional near-infrared spectroscopy and event-related potentials Human Brain Mapping 2008 29 28 35 10.1002/hbm.20368 17315227 PMC6870965

[B8] Minati L Jones CL Gray MA Medford N Harrison NA Critchley HD Emotional modulation of visual cortex activity: a functional near-infrared spectroscopy study Neuroreport 2009 20 1344 50 10.1097/WNR.0b013e328330c751 19738501 PMC2892113

[B9] Lang PJ Bradley MM Cuthbert BN International Affective Picture System (IAPS): Technical Manual and Affective Ratings 1997 Gainesville: University of Florida, Center for Research in Psychophysiology

[B10] Junghöfer M Bradley MM Elbert TR Lang PJ Fleeting images: a new look at early emotion discrimination Psychophysiology 2001 38 175 178 10.1017/S0048577201000762 11347862

[B11] Schupp HT Cuthbert BN Bradley MM Cacioppo JT Ito T Lang PJ Affective picture processing: the late positive potential is modulated by motivational relevance Psychophysiology 2000 37 257 261 10.1017/S0048577200001530 10731776

[B12] Schupp HT Junghöfer M Weike AI Hamm AO Attention and emotion: an ERP analysis of facilitated emotional stimulus processing Neuroreport 2003 14 1107 1110 10.1097/00001756-200306110-00002 12821791

[B13] Keil A Gruber T Müller MM Moratti S Stolarova M Bradley MM Lang PJ Early modulation of visual perception by emotional arousal: evidence from steady-state visual evoked brain potentials Cognitive, Affective & Behavioral Neuroscience 2003 3 195 206 10.3758/cabn.3.3.195 14672156

[B14] Moratti S Keil A Stolarova M Motivated attention in emotional picture processing is reflected by activity modulation in cortical attention networks NeuroImage 2004 21 954 964 10.1016/j.neuroimage.2003.10.030 15006662

[B15] Amaral DG Behniea H Kelly JL Topographic organization of projections from the amygdala to the visual cortex in the macaque monkey Neuroscience 2003 118 1099 1120 10.1016/S0306-4522(02)01001-1 12732254

[B16] LeDoux JE Emotion circuits in the brain Annual Review of Neuroscience 2000 23 155 184 10.1146/annurev.neuro.23.1.155 10845062

[B17] Kissler J Assadollahi R Herbert C Emotional and semantic networks in visual word processing: insights from ERP studies Progress in Brain Research 2006 156 147 183 full_text 17015079 10.1016/S0079-6123(06)56008-X

[B18] Kanske P Kotz SA Concreteness in emotional words: ERP evidence from a hemifield study Brain Research 2007 1148 138 148 10.1016/j.brainres.2007.02.044 17391654

[B19] Herbert C Kissler J Junghöfer M Peyk P Rockstroh B Processing of emotional adjectives: Evidence from startle EMG and ERPs Psychophysiology 2006 43 197 206 10.1111/j.1469-8986.2006.00385.x 16712590

[B20] Herbert C Junghofer M Kissler J Event related potentials to emotional adjectives during reading Psychophysiology 2008 45 487 498 10.1111/j.1469-8986.2007.00638.x 18221445

[B21] Kissler J Herbert C Peyk P Junghofer M Buzzwords: early cortical responses to emotional words during reading Psychological Science 2007 18 475 480 10.1111/j.1467-9280.2007.01924.x 17576257

[B22] Kissler J Herbert C Winkler I Junghofer M Emotion and attention in visual word processing: an ERP study Biological Psychology 2009 80 75 83 10.1016/j.biopsycho.2008.03.004 18439739

[B23] Schacht A Sommer W Time course and task dependence of emotion effects in word processing Cognitive, Affective & Behavioral Neuroscience 2009 9 28 43 10.3758/CABN.9.1.28 19246325

[B24] Fischler I Bradley M Event-related potential studies of language and emotion: words, phrases, and task effects Progress in Brain Research 2006 156 185 203 full_text 17015080 10.1016/S0079-6123(06)56009-1

[B25] Williamson S Harpur TJ Hare RD Abnormal processing of affective words by psychopaths Psychophysiology 1991 28 260 273 10.1111/j.1469-8986.1991.tb02192.x 1946892

[B26] Schapkin SA Gusev AN Kuhl J Categorization of unilaterally presented emotional words: an ERP analysis Acta Neurobiologiae Experimentalis 2000 60 17 28 10769926 10.55782/ane-2000-1321

[B27] Herbert C Kissler J Motivational priming and processing interrupt: Startle reflex modulation during shallow and deep processing of emotional words International Journal of Psychophysiology 2010 76 64 71 10.1016/j.ijpsycho.2010.02.004 20171998

[B28] Isenberg N Silbersweig D Engelien A Emmerich S Malavade K Beattie B Leon AC Stern E Linguistic threat activates the human amygdala Proceedings of the National Academy of Sciences of the United States of America 1999 96 10456 10459 10.1073/pnas.96.18.10456 10468630 PMC17910

[B29] Tabert MH Borod JC Tang CY Lange G Wei TC Johnson R Nusbaum AO Buchsbaum MS Differential amygdala activation during emotional decision and recognition memory tasks using unpleasant words: an fMRI study Neuropsychologia 2001 39 556 573 10.1016/S0028-3932(00)00157-3 11257281

[B30] Herbert C Ethofer T Anders S Junghofer M Wildgruber D Grodd W Kissler J Amygdala activation during reading of emotional adjectives--an advantage for pleasant content Social Cognitive and Affective Neuroscience 2009 4 35 49 10.1093/scan/nsn027 19015080 PMC2656883

[B31] Cheung C Culver JP Takahashi K Greenberg JH Yodh AG In vivo cerebrovascular measurement combining diffuse near-infrared absorption and correlation spectroscopies Physics in Medicine and Biology 2001 46 2053 2065 10.1088/0031-9155/46/8/302 11512610

[B32] Durduran T Yu G Burnett MG Detre JA Greenberg JH Wang J Zhou C Yodh AG Diffuse optical measurement of blood flow, blood oxygenation, and metabolism in a human brain during sensorimotor cortex activation Optics Letters 2004 29 1766 1768 10.1364/OL.29.001766 15352363

[B33] Jaillon F Li J Dietsche G Elbert T Gisler T Activity of the human visual cortex measured non-invasively by diffusing-wave spectroscopy Optics Express 2007 15 6643 6650 10.1364/OE.15.006643 19546974

[B34] Li J Dietsche G Iftime D Skipetrov SE Maret G Elbert T Rockstroh B Gisler T Noninvasive detection of functional brain activity with near-infrared diffusing-wave spectroscopy Journal of Biomedical Optics 2005 10 44002 44002 10.1117/1.2007987 16178636

[B35] Li J Ninck M Koban L Elbert T Kissler J Gisler T Transient functional blood flow change in the human brain measured noninvasively by diffusing-wave spectroscopy Optics Letters 2008 33 2233 2235 10.1364/OL.33.002233 18830362

[B36] Dietsche G Ninck M Ortolf C Li J Jaillon F Gisler T Fiber-based multispeckle detection for time-resolved diffusing-wave spectroscopy: characterization and application to blood flow detection in deep tissue Applied Optics 2007 46 8506 8514 10.1364/AO.46.008506 18071383

[B37] Ito H Takahashi K Hatazawa J Kim SG Kanno I Changes in human regional cerebral blood flow and cerebral blood volume during visual stimulation measured by positron emission tomography Journal of Cerebral Blood Flow and Metabolism 2001 21 608 612 11333371 10.1097/00004647-200105000-00015

[B38] Buxton RB Uludağ K Dubowitz DJ Liu TT Modeling the hemodynamic response to brain activation NeuroImage 2004 23 Suppl 1 S220 233 10.1016/j.neuroimage.2004.07.013 15501093

[B39] Regan D Human Brain Electrophysiology 1989 New York: Elsevier

[B40] Silberstein RB Nunez PL Steady-State Visually Evoked Potentials, Brain Resonances, and Cognitive Processes Neocortical Dynamics and Human EEG Rhythms 1995 New York: Oxford University Press

[B41] Morgan ST Hansen JC Hillyard SA Selective attention to stimulus location modulates the steady-state visual evoked potential Proceedings of the National Academy of Sciences of the United States of America 1996 93 4770 4774 10.1073/pnas.93.10.4770 8643478 PMC39354

[B42] Müller MM Teder-Sälejärvi W Hillyard SA The time course of cortical facilitation during cued shifts of spatial attention Nature Neuroscience 1998 1 631 634 10.1038/2865 10196572

[B43] Chen Y Seth AK Gally JA Edelman GM The power of human brain magnetoencephalographic signals can be modulated up or down by changes in an attentive visual task Proceedings of the National Academy of Sciences of the United States of America 2003 100 3501 3506 10.1073/pnas.0337630100 12626756 PMC152322

[B44] Ding J Sperling G Srinivasan R Attentional modulation of SSVEP power depends on the network tagged by the flicker frequency Cerebral Cortex 2006 16 1016 1029 10.1093/cercor/bhj044 16221931 PMC1880883

[B45] Bradley MM Lang PJ Measuring emotion: the Self-Assessment Manikin and the Semantic Differential Journal of Behavior Therapy and Experimental Psychiatry 1994 25 49 59 10.1016/0005-7916(94)90063-9 7962581

[B46] American Clinical Neurophysiology S Guideline 5: Guidelines for standard electrode position nomenclature Journal of Clinical Neurophysiology 2006 23 107 110 10.1097/00004691-200604000-00006 16612226

[B47] Berg P Scherg M A multiple source approach to the correction of eye artifacts Electroencephalography and Clinical Neurophysiology 1994 90 229 241 10.1016/0013-4694(94)90094-9 7511504

[B48] Muller MM Andersen SK Keil A Time Course of Competition for Visual Processing Resources between Emotional Pictures and Foreground Task Cerebral Cortex 2008 18 1892 1899 10.1093/cercor/bhm215 18063562

[B49] Keil A Macroscopic brain dynamics during verbal and pictorial processing of affective stimuli Progress in Brain Research 2006 156 217 232 full_text 17015082 10.1016/S0079-6123(06)56011-X

[B50] Schupp HT Stockburger J Codispoti M Junghöfer M Weike AI Hamm AO Selective visual attention to emotion The Journal of Neuroscience 2007 27 1082 1089 10.1523/JNEUROSCI.3223-06.2007 17267562 PMC6673176

[B51] Kuchinke L Jacobs AM Grubich C Võ MLH Conrad M Herrmann M Incidental effects of emotional valence in single word processing: an fMRI study NeuroImage 2005 28 1022 1032 10.1016/j.neuroimage.2005.06.050 16084739

[B52] Keil A Moratti S Sabatinelli D Bradley MM Lang PJ Additive effects of emotional content and spatial selective attention on electrocortical facilitation Cerebral Cortex 2005 15 1187 1197 10.1093/cercor/bhi001 15590910

[B53] Kemp AH Silberstein RB Armstrong SM Nathan PJ Gender differences in the cortical electrophysiological processing of visual emotional stimuli NeuroImage 2004 21 632 646 10.1016/j.neuroimage.2003.09.055 14980566

[B54] Vuilleumier P Driver J Modulation of visual processing by attention and emotion: windows on causal interactions between human brain regions Philosophical Transactions of the Royal Society of London Series B, Biological Sciences 2007 362 837 855 10.1098/rstb.2007.2092 17395574 PMC2430001

[B55] Keil A Sabatinelli D Ding M Lang PJ Ihssen N Heim S Re-entrant projections modulate visual cortex in affective perception: evidence from Granger causality analysis Human Brain Mapping 2009 30 532 540 10.1002/hbm.20521 18095279 PMC3622724

[B56] Junghöfer M Schupp HT Stark R Vaitl D Neuroimaging of emotion: empirical effects of proportional global signal scaling in fMRI data analysis NeuroImage 2005 25 520 526 10.1016/j.neuroimage.2004.12.011 15784431

[B57] Sabatinelli D Bradley MM Fitzsimmons JR Lang PJ Parallel amygdala and inferotemporal activation reflect emotional intensity and fear relevance NeuroImage 2005 24 1265 1270 10.1016/j.neuroimage.2004.12.015 15670706

[B58] Sabatinelli D Bradley MM Lang PJ Costa VD Versace F Pleasure rather than salience activates human nucleus accumbens and medial prefrontal cortex Journal of Neurophysiology 2007 98 1374 1379 10.1152/jn.00230.2007 17596422

